# JAK Inhibition as a Potential Treatment Target in Systemic Lupus Erythematosus

**DOI:** 10.31138/mjr.231123.jia

**Published:** 2024-03-30

**Authors:** Georgia-Savina Moysidou, Athanasia Dara

**Affiliations:** 1 National and Kapodistrian University of Athens, Faculty of Medicine, Athens, Greece; Inflammation and Autoimmunity Lab, Biomedical Research Foundation of the Academy of Athens (BRFAA), Athens, Greece,; 2 Fourth Department of Internal Medicine, Hippokration Hospital, Aristotle University of Thessaloniki, Thessaloniki, Greece

**Keywords:** JAK, inhibition, potential, treatment, target, systemic lupus erythematosus

## Abstract

Janus kinase (JAK)/signal transducers and activators of transcription (STATs) are a group of molecules responsible for signal transduction of multiple cytokines and growth factors in different cell types, involved in the maintenance of immune tolerance. Thus, the dysregulation of this pathway plays a crucial role in the pathogenesis of multiple autoimmune, inflammatory, and allergic diseases and is an attractive treatment target. JAK inhibitors (JAKinibs) have been approved in the treatment of multiple autoimmune diseases including rheumatoid arthritis (RA), psoriatic arthritis (PsA) and ankylosing spondylitis (SPA). In SLE, there is a plethora of ongoing trials evaluating their efficacy, with tofacitinib, baricitinib and deucravacitinib showing promising results, without major safety concerns. In this review, we will discuss the rationale of targeting JAKinibs in SLE and summarize the clinical data of efficacy and safety of JAKinibs in SLE patients.

## INTRODUCTION

SLE is a chronic autoimmune disease affecting multiple organs with a variable clinical course and prognosis.^1^ Its pathogenesis is elusive and multifactorial implicating both the innate and adaptive immunity^2^ and is characterised by the aberrant production of several cytokines and autoantibodies. The treatment goal is the attainment of remission or Lupus Low Disease Activity State (LLDAS) which is associated with better outcomes including damage accrual, reduction in the number of flares, glucocorticoids’ withdrawal, and better quality of life.^3^ Combination therapy of immunomodulatory agents as well as glucocorticoids may be necessary in achieving the treatment target. To date, unlike other RMDs where a plethora of targeted therapies is available, there are only two biological therapies - belimumab and anifrolumab – that have been approved for the treatment of SLE.

The last decade, JAKinibs blocking simultaneously one or more Janus kinases, are a valuable treatment option in the therapeutic armamentarium for patients with RMDs, including those with difficult-to-treat^4^ or refractory disease.^5^

Herein, we overview the role of the JAK/STAT pathway in SLE with a focus on ongoing clinical trials of JAKinibs in SLE.

## JAK/STAT PATHWAY PATHOPHYSIOLOGY

The JAK/STAT pathway includes four non-transmembrane tyrosine kinases: the Janus kinases (JAKs) (JAK1, JAK2, JAK3, TYK2) and seven signal transducers and activators of transcription (STATs) (STAT1, STAT2, STAT3, STAT4, STAT5A, STAT5B, STAT6) which are responsible for the signal transduction of over fifty cytokines (including interleukin and interferon members) and growth factors in various cell types regulating gene expression and cell activation, proliferation, and differentiation, playing a crucial role in hematopoiesis, regulation of the immune system, metabolism, and growth.^6^ JAK1, JAK2, and TYK2 are expressed in almost all tissues whereas JAK3 is only expressed in the bone marrow, lymphatic system, endothelial cells, and vascular smooth muscle cells.^7^ The activation of the JAK/STAT pathway is complicated and regulated on multiple levels. The critical step is the activation of JAK upon binding of a ligand to its (type I or II) receptor.^8^ After JAK-mediated phosphorylation at specific receptor tyrosine residues, STATs are recruited to the receptors and become phosphorylated by JAKs.^9^ STATs are subsequently translocated to the nucleus where they bind to members of the gamma-activated site (GAS) family of enhancers, inducing the transcription of target genes.^9^ Aberrant JAK/STAT pathway signaling is prevented by the production of inhibitory proteins acting as checkpoints including the suppressor of cytokine signaling (SOCS) family, or the autoinhibitory mechanism of JH2–JH1 domain interaction.^10^ The breakdown of JAK regulation leads potentially to disease with aberrant activation being linked to tumorigenesis and autoimmunity^11–13^ and loss-of-function mutations being associated with severe combined immune deficiency.^14^

## JAK/STAT PATHWAY AND SLE

The pathogenesis of SLE is complex and characterized by the presence of autoreactive T cells and production of autoantibodies by B cells, with the implication of numerous circulating pro-inflammatory cytokines including interleukin (IL)-2, IL-6, IL-10, IL-12/IL-23, IL-19, IL-20, IL-22, and interferon (IFN)-α, IFN-γ.^15,16^ IFNa is a key cytokine in SLE pathogenesis, and interferon-inducible gene expression is associated with disease activity and lupus nephritis^17,18^ whereas the persistence of high levels of ultrasensible IFN-a is related with a higher risk of relapse in patients with quiescent SLE .^19^

The JAK/STAT pathway is implicated in SLE pathogenesis on multiple levels. First, IFN type I and type II (as well as multiple other proinflammatory cytokines) bind to JAK receptors in order to activate intracellular signals and exert their functions.^20^ Second, there is growing evidence that the JAK/STAT pathway may regulate IFN- regulatory factor (IRF)-related genes.^21^ A study of the relation between type I–II IFN trigger activation of the JAK/STAT pathway in 22 SLE patients indicated that the overexpression of STAT1 by B-cells correlated with lupus activity. More specifically, STAT1 appeared to be activated following exposure to IFNa in SLE plasmablasts. Therefore, the use of JAK inhibitors to prevent STAT1 expression and B cell induction in SLE patients would impede two major pathogenic pathways.^22^

Furthermore, several polymorphisms in the STATs have been related to SLE susceptibility according to genome-wide association studies.^23,24^ Importantly, STAT4 is one of the most dominant risk alleles in SLE^25^ and is related with renal disease and anti-ds DNA positivity.^26^ On the other hand, defects in the deactivation of the JAK/STAT signaling have also been linked to SLE. In the PBMCs from 34 SLE patients the SOCS1 mRNA expression and protein level was reduced compared to 34 healthy controls,^27^ whereas SOCS1 mRNA expression was inversely correlated with SLE disease activity.^27^ Down-regulation of SOCS1 has also been linked to lupus nephritis in SLE patients and murine models and seems to ultimately contribute to renal inflammation and fibrosis^28,29^ but also with numerous other manifestations including skin and central nervous system.^30^ Interestingly, treatment with corticosteroids to human PBMCs leads to upregulation of SOCS1 message in a dose- and time-dependent manner.^31^

## JAK INHIBITION IN SLE: FROM MURINE MODELS TO HUMAN STUDIES

### Murine lupus: blocking JAKs and STATs

There are multiple experimental studies evaluating the role of the JAK/STAT pathway as a potential treatment target, focusing mostly on cutaneous lupus, lupus nephritis and serological activity. In a study in MRL/lpr mice, treatment with a JAK2-STAT1 inhibitor, led to an improvement in nephritis, sialadenitis and anti-ds DNA levels.^32^ In another murine study, JAK2 selective inhibition with CEP-33779, led to extended survival, reduced splenomegaly and lymphomegaly, and decreased several cytokines including IL-12, IL-17A, IFN-α, IL-1β, and TNF-α in MRL/lpr or BWF1 mice with established SLE or lupus nephritis.^33^ Importantly, treatment with CEP-33779 had a significant disease-modifying effect and was able to mitigate several immune parameters associated with SLE disease progression, including the prevention and treatment of mice with lupus nephritis.^33^ Tofacitinib, a JAK1 and JAK3 inhibitor used in the treatment of multiple autoimmune diseases, has also been evaluated in 3 murine models. Mice treated with tofacitinib had reduced proteinuria, improved renal function and histological lesions of the kidney, decreased expression of several IFN-signature genes, and improved vascular function.^34–36^ Ruxolitinib, a JAK1 and JAK2 inhibitor approved in the treatment of myelofibrosis, attenuated the development of severe skin lesions in the MRL/lpr mice, reduced lesion severity scores by week 4 and led to a significant reduction in epidermal hyperplasia and inflammatory cell infiltration.^37^ STAT3 is another attractive treatment target as its activation is highly correlated with different types of glomerulonephritis and is implicated in glomerular and tubulointerstitial cell proliferation, interstitial fibrosis, and the level of renal injury.^38^ Simultaneous blocking of JAK1 and STAT3 by CDDO-Me (C-28 methyl ester of 2-cyano-3,12-dioxoolean- 1,9-dien-28-oic acid) showed promising results in murine models with established LN and prevented the development of LN in two murine models.^39^ On the other hand, mice treated with compound S31-201 (Stattic), a selective STAT3 inhibitor, had a lesser degree of tubule injury, inflammation, and interstitial fibrosis,^40^ whereas Edwards et al. showed that treatment with Stattic delayed onset of proteinuria by 3 weeks compared with controls and lowered levels of anti-dsDNA antibodies and inflammatory cytokines.^41^

TYK2-deficient human T cells exhibit impaired responses to IL-12, IL-23, IFN-α, and IL-10^42^ whereas Tyk2 dysfunction has been related to the prevention of immune- mediated inflammatory diseases including psoriasis, psoriatic arthritis and vitiligo^43,44^ without immunodeficiency.^45^ In SLE, TYK2 is implicated in the increased autophagy activity by B cells^46^ and NPSLE.^47^

### Clinical trials and real-world data

#### Tofacitinib

After the encouraging results of tofacitinib in SLE murine models, its effectiveness has been evaluated in a phase I double-blinded trial including 30 SLE patients.^48^ Patients were stratified based on the presence of the STAT4 risk allele, which may correlate with increased production of type I IFNs in PBMCs and thus with a more severe phenotype and increased risk of cardiovascular disease in SLE patients. In this trial, tofacitinib was found to be not only safe but also improve cardiometabolic and immunologic parameters including high-density lipoprotein cholesterol levels, decreasing type I IFN signature and circulating neutrophil extracellular traps (NETs), especially in the subgroup of patients with the STAT4 risk allele.^48^ In clinical practice, tofacitinib led to rapid remission seven out of ten patients with active skin and/or musculoskeletal disease.^49^ Interestingly, tofacitinib led to significant clinical improvement in 3 patients with recalcitrant cutaneous SLE.^50^

#### Baricitinib

Baricitinib, an oral selective inhibitor of JAK1 and JAK2, proved to be safe and effective at the dose of 4 mg in the resolution of arthritis or rash at week 24 in combination with standard of care in a phase 2 RCT of 314 SLE patients.^51^ In this trial, baricitinib reduced anti-dsDNA titres, and other pro-inflammatory cytokines.^52,53^ In SLE-BRAVE 1, a phase 3 RCT, 760 patients were randomly assigned to receive baricitinib 4mg, 2mg, or placebo.^54^

The primary endpoint, SLE Responder Index 4 (SRI-4) at week 52, was met in the baricitinib 4mg group.^54^ These results were not replicated in SLE-BRAVE 2, another phase 3 RCT.^55^ In this trial, treatment with baricitinib did not meet neither the primary efficacy endpoint of SRI-4 at week 42, nor the major secondary endpoints including glucocorticoid tapering and time to first flare.^55^ Analysis of the above-mentioned studies indicates that while neither the 2mg nor the 4 mg baricitinib dose correlated with a significant increase in the odds of achieving an LLDAS response, the 4 mg dose was associated with a significant increase in the odds of an SRI-4 response, compared to placebo.^56^

#### Solcitinib

Solcitinib, a selective JAK 1 inhibitor, was evaluated in a phase II study in patients with active, extra-renal SLE.^57^ In this RCT, patients received solcitinib or placebo twice daily for 12 weeks. Due to absence of significant effect on one of the primary endpoints, mean IFN transcriptional biomarker expression, the study was prematurely terminated after the recruitment of 50 patients.^57^ Of note, six patients had elevated liver enzymes of whom one confirmed and one suspected case of drug reaction with eosinophilia and systemic symptoms.^57^

#### Filgotinib

Filgotinib, another JAK1 inhibitor has been assessed in a phase 2 trial of patients with cutaneous lupus erythematosus (CLE).^58^ In this trial, the primary endpoint of change from baseline in Cutaneous Lupus Erythematosus Disease Area and Severity Index Activity (CLASI-A) score at week 12 was not met. Of note, 1 serious adverse event was reported in the filgotinib group. However, filgotinib showed promising results in a small study of nine patients with class V LN.^59^ In this study, only four patients in the filgotinib group completed week 16; with a median reduction of 50.7% in 24-hour urine protein.^59^

#### Deucracitinib

Deucracitinib is the first FDA-approved oral TYK2 inhibitor for the treatment of moderate to severe plaque psoriasis.^60^ In SLE, there are promising results from a phase 2 study including 363 patients with active SLE.^61^ In this study, patients receiving 3 mg of deucracitinib twice daily met the primary endpoint, SRI-4 at week 32,^61^ while deucracitinib treated patients had higher frequencies of attainment of LLDAS, BICLA response and CLASI-50 and improvement in arthritis at week 48.^61^

#### Topical treatment: ruxolitinib

Ruxolitinib is effective in the treatment of several immunologic skin disorders, including alopecia areata.^62^ The efficacy of ruxolitinib cream will be assessed in a phase 2 trial of patients with active discoid lupus erythematosus.^63^

#### Upadacitinib

Upadacitinib is a selective JAK1 inhibitor used in the treatment of autoimmune disorders including RA, atopic dermatitis, psoriatic arthritis, and ulcerative colitis.^64^ In a phase II, double-blind trial of 341 SLE patients receiving elsubrutinib/upadacitinib combination therapy, upadacitinib monotherapy or placebo both therapeutic regimens significantly improved disease activity and risk of relapse.^65^ Both primary and key secondary endpoints were achieved whereas anti-double stranded DNA antibodies also significantly decreased in response to treatment. No safety concerns were raised, as no malignancies or VTEs were reported, and the 3 non-fatal CV events were not attributed to the treatment.^65^ Another case study of a 26-year-old female CLE patient receiving upadacitinib, demonstrated significant improvement of lesions using the Revised Cutaneous Lupus Erythematosus Disease Area and Severity Index.^66^

## SAFETY

Important concerns on the safety of JAKis have been raised after the post hoc analysis of the ORAL surveillance, a randomised, open-label, noninferiority, post-authorisation, safety end-point trial.^67^ ORAL surveillance showed that RA patients with cardiovascular risk factors treated with tofacitinib had a higher likelihood of developing a cardiovascular event, including myocardial infarction, stroke, and death due to cardiovascular disease, compared to those treated with anti-TNF.^67^ Treatment with tofacitinib was also related to higher incidence of adjudicated opportunistic infections (including herpes zoster and tuberculosis), and adjudicated nonmelanoma skin cancer.^67^ It is speculated that the greater inhibition of JAK2, achieved with higher doses of tofacitinib and baricitinib, might be the underlying mechanism responsible for the increased risk of a thromboembolic event.^67^ However, a systematic review and meta-analysis of 29 RCTs did not show augmented risk of CVEs and all-cause mortality in patients with immune-mediated inflammatory diseases on tofacitinib treatment in a short-term perspective.^68^ Data from a ‘real-world evidence (RWE) cohort’ including 12 852 RA, tofacitinib-treated patients did not find statistically significant evidence for an increased risk of cardiovascular outcomes.^69^ Regarding the risk of serious infection, both Wallace et al. and Petri et al. reported serious adverse events and major infections in patients receiving either 2mg or 4 mg of baricitinib. No deaths attributed to baricitinib use were reported in the aforementioned studies.^51,55^

## CONCLUDING REMARKS

Despite recent advances in the treatment of rheumatic diseases there are substantial unmet needs in the management of SLE patients, mainly due to the heterogenicity and the complexity of the disease and problems in study design including too strict endpoints. JAKis, are nowadays used in the treatment of multiple autoimmune diseases, proving their efficacy even in patients with refractory disease^5,49^ due to their potential of simultaneous inhibition of multiple cytokines. In SLE, tofacitinib, baricitinib, and deucracitinib have shown encouraging results with an acceptable safety profile. Larger trials and real-world data are necessary in order to evaluate the efficacy and safety of JAKis especially in patients with severe manifestations including LN and NPSLE.

**Table 1.** JAK inhibitor clinical trials of efficacy and safety.

**Reference**	**Compound**	**Target**	**Study population**	**Phase**	**Key Results**	**Safety concerns**
Hasni et al. (2021)	Tofacitinib (5 mg twice daily)	JAK1/JAK3	30 SLE patients	Ib/IIa	Improvement of cardiometabolic and immunologic parameters	No
Wallace et al. (2018)	Baricitinib (2 mg or 4mg)	JAK1 /JAK2	314 SLE patients	II	Improvement of arthritis, rash and anti-dsDNA titres at week 24	Serious adverse events in 10 patients receiving 4 mg dose and 11 receiving 2 mg dose Serious infections in 6 patients with 4 mg dose and 2 with 2 mg dose No deaths reported
Dörner et al. (2022)	Baricitinib (2mg and 4mg)	JAK1 /JAK2	239 SLE patients	II	Downregulation of key cytokines CD137, PD- L1, IL-6 and IL-12β at week 12	No
Morand et al. (2023)	Baricitinib (4 mg, 2 mg once daily)	JAK1/JAK2	760 SLE patients	III	SRl-4 response at 52 weeks met in the baricitinib 4 mg group	No
Petri et al. (2023)	Baricitinib (4 mg or 2 mg once daily)	JAK1/JAK2	775 SLE patients	III	SRI-4 at week 52 not met	4mg dose: serious adverse effects in 11% of participants 2mg dose: serious adverse effects in 13% of participants
Kahl et al. (2016)	GSK2586184 (50–400 mg twice daily)	JAK1	50 SLE patients	II	No significant effect on mean interferon transcriptional biomarker expression at 12 weeks Study declared futile	Elevation of liver enzymes in 6 patients
Werth et al. (2022)	Filgotinib (FIL)(200 mg once daily)/lanraplenib (LANRA)(30 mg once daily)	JAK1/SYK	47 CLE patients	II	CLASI-A score at week 12 not met	Two serious AEs reported in the LANRA group and one in the FIL group
Baker et al. (2020)	Filgotinib (FIL) ( 30 mg once daily)/lanraplenib (LANRA) (200mg once daily)	JAK1/SYK	9 LMN patients	II	50.7% reduction in 24- hour urine protein at week 16 in 4 patients of the FIL arm completing week	No
Morand et al. (2023)	Deucravacitinib (3 mg twice daily, 6 mg twice daily or 12 mg once daily)	TYK2	363 SLE patients	II	Higher SRI-4, BICLA, CLASl-50, LLDAS response rates at week 32	No
Merrill et al. (2023)	Elsubrutinib (60 mg) and Upadacitinib (30mg), or Elsubrutinib (60 mg) and Upadacitinib (15mg), upadacitinib (30mg), or Elsubrutinib (60mg)	BTK /JAK1	341 patients	II	SRI-4 response by week 24 met Reduction of overall flares and time to first flare	No

## References

[1] Tselios K Gladman DD Touma Z Su J Anderson N Urowitz MB. Disease course patterns in systemic lupus erythematosus. Lupus 2019 Jan;28(1):114–22. 30526328 10.1177/0961203318817132

[2] Wahren-Herlenius M Dorner T. Immunopathogenic mechanisms of systemic autoimmune disease. Lancet 2013;382(9894): 819–31. 23993191 10.1016/S0140-6736(13)60954-X

[3] Rios-Garces R Espinosa G van Vollenhoven R Cervera R. Treat-to-target in systemic lupus erythematosus: Where are we? Eur J Intern. Med. 2020;74:29–34. 10.1016/j.ejim.2020.01.018 32014364

[4] Ochi S Sonomoto K Nakayamada S Tanaka Y. Preferable outcome of Janus kinase inhibitors for a group of difficult-to-treat rheumatoid arthritis patients: from the FIRST Registry. Arthritis Res Ther 2022;24:61. 35232462 10.1186/s13075-022-02744-7 PMC8886884

[5] Zhang J Sun L Shi X Li S Liu C Li X Janus kinase inhibitor, tofacitinib, in refractory juvenile dermatomyositis: a retrospective multi-central study in China. Arthritis Res Ther 2023;25:204. 37853451 10.1186/s13075-023-03170-z PMC10583374

[6] Reddy D Kumavath R Ghosh P Barh D. Lanatoside C induces G2/M cell cycle arrest and suppresses cancer cell growth by attenuating MAPK, Wnt, JAK-STAT, and PI3K/AKT/mTOR signaling pathways. Biomolecules 2019;9(12):792. 31783627 10.3390/biom9120792 PMC6995510

[7] Verbsky JW Bach EA Fang YF Yang L Randolph DA Fields LE. Expression of Janus kinase 3 in human endothelial and other nonlymphoid and non-myeloid cells. J Biol Chem 1996;271:13976–80. 8662778 10.1074/jbc.271.24.13976

[8] Seif F Khoshmirsafa M Aazami H Mohsenzadegan M Sedighi G Bahar M. The role of JAK-STAT signaling pathway and its regulators in the fate of T helper cells. Cell Commun Signal 2017;15:23. 28637459 10.1186/s12964-017-0177-y PMC5480189

[9] Alunno A Padjen I Fanouriakis A Boumpas DT. Pathogenic and therapeutic relevance of JAK/STAT signaling in systemic lupus Erythematosus: integration of distinct inflammatory pathways and the Prospect of their inhibition with an Oral agent. Cells 2019;8:898. doi: 10.3390/cells8080898 31443172 PMC6721755

[10] Hammarén HM Virtanen AT Raivola J Silvennoinen O. The regulation of JAKs in cytokine signaling and its breakdown in disease. Cytokine 2019;118:48–63. 29685781 10.1016/j.cyto.2018.03.041

[11] Thomas S Snowden J Zeidler M Danson S. The role of JAK/STAT signaling in the pathogenesis, prognosis and treatment of solid tumours. Br J Cancer 2015;113, 365–71. 26151455 10.1038/bjc.2015.233 PMC4522639

[12] O’Shea JJ Schwartz DM Villarino AV Gadina M McInnes IB Laurence A. The JAK–STAT pathway: Impact on human disease and therapeutic intervention. Annu Rev Med 2015:66:311–28. 25587654 10.1146/annurev-med-051113-024537 PMC5634336

[13] Milner JD Vogel TP Forbes L Ma CA Stray-Pedersen A Niemela JE Early-onset lymphoproliferation and autoimmunity caused by germline STAT3 gain-of-function mutations. Blood 2015;125:591–9. 25359994 10.1182/blood-2014-09-602763 PMC4304103

[14] Macchi P Villa A Giliani S Sacco MG Frattini A Porta F Mutations of jak-3 gene in patients with autosomal severe combined immune deficiency (SCID). Nature 1995;377:65–8. 7659163 10.1038/377065a0

[15] Ohl K Tenbrock K. Inflammatory cytokines in systemic lupus erythematosus. J Biomed Biotechnol 2011;432595:1–14. 10.1155/2011/432595 PMC3196871 22028588

[16] Liu Z Davidson A. Taming lupus — a new understanding of pathogenesis is leading to clinical advances. Nat Med 2012;18:871–82. 22674006 10.1038/nm.2752 PMC3607103

[17] Feng X Wu H Grossman JM Hanvivadhanakul P FitzGerald JD Park GS Association of increased interferon-inducible gene expression with disease activity and lupus nephritis in patients with systemic lupus erythematosus. Arthritis Rheum 2006 Sep;54(9):2951–62. 16947629 10.1002/art.22044

[18] Bengtsson AA Sturfelt G Truedsson L Blomberg J Alm G Vallin H Activation of type I interferon system in systemic lupus erythematosus correlates with disease activity but not with antiretroviral antibodies. Lupus 2000;9(9):664–71. 11199920 10.1191/096120300674499064

[19] Mathian A Mouries-Martin S Dorgham K Devilliers H Yssel H Garrido Castillo L Ultrasensitive serum interferon–α quantification during SLE remission identifies patients at risk for relapse. Ann Rheum Dis 2019 Dec;78(12):1669–76. 31570366 10.1136/annrheumdis-2019-215571

[20] Lin CM Cooles FA Isaacs JD. Basic mechanisms of JAK inhibition. Mediterr J Rheumatol 2020;31:100–4. 32676567 10.31138/mjr.31.1.100 PMC7361186

[21] Kawasaki M Fujishiro M Yamaguchi A Nozawa K Kaneko H Takasaki Y Possible roles of the JAK/STAT pathways in the regulation of T-cell interferon related genes insystemic lupus erythematosus. Lupus 2011;20:1231–9. 21980035 10.1177/0961203311409963

[22] Aue A Szelinski F Weißenberg SY Wiedemann A Rose T Lino AC Elevated STAT1 expression but not phosphorylation in lupus B cells correlates with disease activity and increased plasmablast susceptibility. Rheumatology (Oxford) 2020 Nov 1;59(11):3435–3442. 32357246 10.1093/rheumatology/keaa187

[23] Alarcón-Riquelme ME Ziegler JT Molineros J Howard TD Moreno-Estrada A Sánchez-Rodríguez E Genome-Wide Association Study in an Amerindian Ancestry Population Reveals Novel Systemic Lupus Erythematosus Risk Loci and the Role of European Admixture. Arthritis Rheumatol 2016 Apr;68(4):932–43. 26606652 10.1002/art.39504 PMC4829354

[24] heng J Yin J Huang R Petersen F Yu X. Meta-analysis reveals an association of STAT4 polymorphisms with systemic autoimmune disorders and anti-dsDNA antibody. Hum Immunol 2013;74:986–92. 23628400 10.1016/j.humimm.2013.04.034

[25] Kariuki SN Kirou KA MacDermott EJ Barillas-Arias L Crow MK Niewold TB. Cutting edge: autoimmune disease risk variant of STAT4 confers increased sensitivity to IFN-alpha in lupus patients in vivo. J Immunol 2009;182:34–8. 19109131 10.4049/jimmunol.182.1.34 PMC2716754

[26] Mok CC. The Jakinibs in systemic lupus erythematosus: progress and prospects. Expert Opin Investig Drugs 2019 Jan;28(1):85–92. 10.1080/13543784.2019.1551358 30462559

[27] Qiu LJ Xu K Liang Y Cen H Zhang M Wen PF Decreased SOCS1 mRNA expression levels in peripheral blood mononuclear cells from patients with systemic lupus erythematosus in a Chinese population. Clin Exp Med. 2015 Aug;15(3):261–7 25330931 10.1007/s10238-014-0309-2

[28] Dong J Wang QX Zhou CY Ma XF Zhang YC. Activation of the STAT1 signalling pathway in lupus nephritis in MRL/lpr mice. Lupus 2007;16:101–9. 17402366 10.1177/0961203306075383

[29] Wang P Yang J Tong F Duan Z Liu X Xia L Anti-Double-Stranded DNA IgG Participates in Renal Fibrosis through Suppressing the Suppressor of Cytokine Signaling 1 Signals. Front Immunol 2017 May 31;8:610. doi: 10.3389/fimmu.2017.00610. eCollection 2017. 28620377 PMC5449454

[30] Wang H Wang J Xia Y. Defective Suppressor of Cytokine Signaling 1 Signaling Contributes to the Pathogenesis of Systemic Lupus Erythematosus. Front Immunol 2017 Oct 16;8:1292. 29085365 10.3389/fimmu.2017.01292 PMC5650678

[31] ukka-Ganesh B Larkin J 3rd. Therapeutic Potential for Targeting the Suppressor of Cytokine Signalling-1 Pathway for the Treatment of SLE. Scand J Immunol 2016 Nov;84(5):299–309. 27781323 10.1111/sji.12475 PMC5131794

[32] Wang S Yang N Zhang L Huang B Tan H Liang Y. Jak/STAT signaling is involved in the inflammatory infiltration of the kidneys in MRL/lpr mice. Lupus (2010) 19:1171–80. 20501525 10.1177/0961203310367660

[33] Lu LD Stump KL Wallace NH Dobrzanski P Serdikoff C Gingrich DE Depletion of autoreactive plasma cells and treatment of lupus nephritis in mice using CEP-33779, a novel, orally active, selective inhibitor of JAK2. J Immunol 2011 Oct 1;187(7):3840–53. 21880982 10.4049/jimmunol.1101228

[34] Ripoll È de Ramon L Draibe Bordignon J Merino A Bolaños N Goma M JAK3-STAT pathway blocking benefits in experimental lupus nephritis. Arthritis Res Ther 2016;18:134. 27278657 10.1186/s13075-016-1034-x PMC4898357

[35] Ikeda K Hayakawa K Fujishiro M Kawasaki M Hirai T Tsushima H JAK inhibitor has the amelioration effect in lupus-prone mice: The involvement of IFN signature gene downregulation. BMC Immunol 2017;18:41. 28830352 10.1186/s12865-017-0225-9 PMC5568047

[36] Furumoto Y Smith CK Blanco L Zhao W Brooks SR Thacker SG Tofacitinib Ameliorates Murine Lupus and Its Associated Vascular Dysfunction. Arthritis Rheumatol 2017;69:148–60. 27429362 10.1002/art.39818 PMC5195893

[37] Chan ES Herlitz LC Jabbari A. Ruxolitinib Attenuates Cutaneous Lupus Development in a Mouse Lupus Model. J Investig Dermatol 2015;135:1912–5. 25789705 10.1038/jid.2015.107

[38] Arakawa T Masaki T Hirai T Doi S Kuratsune M Arihiro K Activation of signal transducer and activator of transcription 3 correlates with cell proliferation and renal injury in human glomerulonephritis. Nephrol Dial Transplant 2008;23: 3418–26. 18556750 10.1093/ndt/gfn314

[39] Wu T Ye Y Min SY Zhu J Khobahy E Zhou J Prevention of murine lupus nephritis by targeting multiple signaling axes and oxidative stress using a synthetic triterpenoid. Arthritis Rheumatol 2014;66:3129–9. 25047252 10.1002/art.38782 PMC4840107

[40] Du Y Zhang W Liu S Feng X Gao F Liu Q. S3I-201 ameliorates tubulointerstitial lesion of the kidneys in MRL/lpr mice. Biochem Biophys Res Commun 2018;503:177–80. 29885836 10.1016/j.bbrc.2018.05.207

[41] Edwards LJ Mizui M Kyttaris V. Signal transducer and activator of transcription (STAT) 3 inhibition delays the onset of lupus nephritis in MRL/lpr mice. Clin Immunol 2015;158:221–30. 25869298 10.1016/j.clim.2015.04.004 PMC4465043

[42] Kreins AY Ciancanelli MJ Okada S Kong X-F Ramírez-Alejo N Kilic SS Human TYK2 deficiency: mycobacterial and viral infections without hyper-IgE syndrome. J Exp Med 2015;212:1641–62. 26304966 10.1084/jem.20140280 PMC4577846

[43] Garcia-Melendo C Cubiró X Puig L. Janus Kinase Inhibitors in Dermatology: Part 1—General Considerations and Applications in Vitiligo and Alopecia Areata. Actas Dermo-Sifiliográficas Engl Ed 2021;112:503–15. 10.1016/j.adengl.2021.05.008 34030992

[44] Garcia-Melendo C Cubiró X Puig L. Janus Kinase Inhibitors in Dermatology: Part 2: Applications in Psoriasis, Atopic Dermatitis, and Other Dermatoses. Actas Dermo-Sifiliográficas Engl Ed 2021;112:586–600. 10.1016/j.adengl.2021.05.008 34030992

[45] Burke JR Cheng L Gillooly KM Strnad J Zupa-Fernandez A Catlett IM Autoimmune pathways in mice and humans are blocked by pharmacological stabilization of the TYK2 pseudokinase domain. Sci Transl Med 2019 Jul 24;11(502):eaaw1736. 31341059 10.1126/scitranslmed.aaw1736

[46] Dong G You M Fan H Ding L Sun L Hou Y. STS-1 promotes IFN-α induced autophagy by activating the JAK1-STAT1 signaling pathway in B cells. Eur J Immunol 2015;45:2377–88. 25959715 10.1002/eji.201445349

[47] Nikolopoulos D Manolakou T Polissidis A Filia A Bertsias G Koutmani Y Microglia activation in the presence of intact blood-brain barrier and disruption of hippocampal neurogenesis via IL-6 and IL-18 mediate early diffuse neuropsychiatric lupus. Ann Rheum Dis 2023;82:646–57. 36898766 10.1136/ard-2022-223506 PMC10176423

[48] Hasni SA Gupta S Davis M Poncio E Temesgen-Oyelakin Y Carlucci PM Phase 1 double-blind randomized safety trial of the Janus kinase inhibitor tofacitinib in systemic lupus erythematosus. Nat Commun 2021;12:3391. 34099646 10.1038/s41467-021-23361-z PMC8185103

[49] You H Zhang G Wang Q Zhang S Zhao J Tian X Successful treatment of arthritis and rash with tofacitinib in systemic lupus erythematosus: the experience from a single Centre. Ann Rheum Dis 2019;78:1441–3. 31005902 10.1136/annrheumdis-2019-215455

[50] Bonnardeaux E Dutz JP. Oral tofacitinib citrate for recalcitrant cutaneous lupus. JAAD Case Rep 2002 Feb;20:61–4. 10.1016/j.jdcr.2021.09.030 PMC8792392 35118185

[51] Wallace DJ Furie RA Tanaka Y Kalunian KC Mosca M Petri MA Baricitinib for systemic lupus erythematosus: a double-blind, randomised, placebo-controlled, phase 2 trial. Lancet 2018;392:222–31. doi: 10.1016/S0140-6736(18)31363-1 30043749

[52] Dörner T van Vollenhoven RF Doria A Jia B Ross Terres JA Silk ME Baricitinib decreases anti-dsDNA in patients with systemic lupus erythematosus: results from a phase II double-blind, randomized, placebo-controlled trial. Arthritis Res Ther 2022;24:112. 35578304 10.1186/s13075-022-02794-x PMC9109322

[53] Dörner T Tanaka Y Dow ER Koch AE Silk M Ross Terres JA Mechanism of action of baricitinib and identification of biomarkers and key immune pathways in patients with active systemic lupus erythematosus. Ann Rheum Dis 2022;81:1267–72. 35609978 10.1136/annrheumdis-2022-222335 PMC9380497

[54] Morand EF Vital EM Petri M van Vollenhoven R Wallace DJ Mosca M Baricitinib for systemic lupus erythematosus: a double-blind, randomised, placebo-controlled, phase 3 trial (SLE-BRAVE-I). Lancet 2023;401:1001–10. doi: 10.1016/S0140-6736(22)02607-1 36848918

[55] Petri M Bruce IN Dörner T Tanaka Y Morand EF Kalunian KC Baricitinib for systemic lupus erythematosus: a double-blind, randomised, placebo-controlled, phase 3 trial (SLE-BRAVE-II). Lancet 2023;401:1011–9. doi: 10.1016/S0140-6736(22)02546-6 36848919

[56] Patoulias D Dimosiari A Dimitroulas T. Baricitinib for systemic lupus erythematosus: not enough, or not enough evidence? Clin Exp Rheumatol 2023 Nov;41(11):2339–40. 37279150 10.55563/clinexprheumatol/0of42a

[57] Kahl L Patel J Layton M Binks M Hicks K Leon G JAK115919 Study Team. Safety, tolerability, efficacy and pharmacodynamics of the selective JAK1 inhibitor GSK2586184 in patients with systemic lupus erythematosus. Lupus 2016 Nov;25(13):1420–30. 27055521 10.1177/0961203316640910

[58] Werth VP Fleischmann R Robern M Touma Z Tiamiyu I Gurtovaya O Filgotinib or Lanraplenib in Moderate to Severe Cutaneous Lupus Erythematosus: A Phase 2, Randomised, Double-Blind, Placebo-Controlled Study. Rheumatology (Oxford) 2021 Sep 8;keab685. 10.1093/rheumatology/keab685 PMC9157055 34498056

[59] Baker M Chaichian Y Genovese M Derebail V Rao P Chatham W Phase II, randomised, double-blind, multicentre study evaluating the safety and efficacy of filgotinib and lanraplenib in patients with lupus membranous nephropathy. RMD Open 2020 Dec;6(3):e001490. 33380521 10.1136/rmdopen-2020-001490 PMC7780527

[60] Truong TM Pathak GN Signal A Taranto V Rao BK. Deucracitinib: The first FDA-Approved oral TYK2 Inhibitor for Moderate to Severe Plaque Psoriasis. Ann Pharmacother 2023 Jun 21:10.600/280231153863. 37341177

[61] Morand E Pike M Merrill JT van Vollenhoven R Werth VP Hobar C Deucravacitinib, a tyrosine kinase 2 inhibitor, in systemic lupus Erythematosus: a phase II, randomized, double-blind. Placebo-Controlled Trial. Arthritis Rheumatol 2023;75:242–52. 36369798 10.1002/art.42391 PMC10100399

[62] Xing L Dai Z Jabbari A Cerise JE Higgins CA Gong W Alopecia areata is driven by cytotoxic T lymphocytes and is reversed by JAK inhibition. Nat Med 2014;20(9):1043–9. 25129481 10.1038/nm.3645 PMC4362521

[63] Study of Ruxolitinib Cream for the Treatment of Discoid Lupus Erythematosus; Available from: https://clinicaltrials.gov/ct2/show/NCT04908280.

[64] Burmester GR Cohen SB Winthrop KL Nash P Irvine AD Deodhar A Safety profile of upadacitinib over 15 000 patient-years across rheumatoid arthritis, psoriatic arthritis, ankylosing spondylitis and atopic dermatitis. RMD Open 2023 Feb;9(1):e002735. 36754548 10.1136/rmdopen-2022-002735 PMC9923346

[65] Merrill JT Tanaka Y D’Cruz D Vila-Rivera K Siri D Zeng X Efficacy and Safety of ABBV-599 High Dose (Elsubrutinib 60 mg and Upadacitinib 30 mg) and Upadacitinib Monotherapy for the Treatment of Systemic Lupus Erythematosus: A Phase 2, Double-blind, Placebo-controlled Trial [abstract]. Ann Rheum Dis 2023;75 (suppl 9).

[66] Hu W Zhang S Lian C. Treatment of Discoid Lupus Erythematosus with Upadacitinib: A Case Report. Clin Cosmet Investig Dermatol 2023 Oct 9;16:2793–800. 10.2147/CCID.S419344 PMC10573460 37841060

[67] Ytterberg SR Bhatt DL Mikuls TR Koch GG Fleischmann R Rivas JL Cardiovascular and Cancer risk with Tofacitinib in rheumatoid arthritis. N Engl J Med 2022;386:316–26. doi: 10.1056/NEJMoa2109927 35081280

[68] Xie W Xiao S Huang Y Sun X Zhang Z. Effect of tofacitinib on cardiovascular events and all-cause mortality in patients with immune-mediated inflammatory diseases: a systematic review and meta-analysis of randomized controlled trials. Ther Adv Musculoskelet Dis 2019;11:1759720X1989549. 10.1177/1759720X19895492 PMC6918042 31897092

[69] Khosrow-Khavar F Kim SC Lee H Lee SB Desai RJ. Tofacitinib and risk of cardiovascular outcomes: results from the Safety of TofAcitinib in Routine care patients with Rheumatoid Arthritis (STAR-RA) study. Ann Rheum Dis 2022;81:798–804. 35027405 10.1136/annrheumdis-2021-221915 PMC9117457

